# Tricuspid surgery at the time of LVAD implant: A critique

**DOI:** 10.3389/fcvm.2022.1056414

**Published:** 2022-11-21

**Authors:** Charles Hoopes

**Affiliations:** Cardiothoracic Surgery, University of Alabama at Birmingham, Birmingham, AL, United States

**Keywords:** tricuspid valve, left ventricular assist device (LVAD), annuloplasty, right ventricular dysfunction, heart failure

## Abstract

Tricuspid regurgitation (TR) is a common finding in patients with end stage heart failure referred for implantation of left ventricular assist devices. While functional TR frequently resolves after left ventricular unloading, patients with residual and progressive TR demonstrate increased rates of RV dysfunction and poor survival. Criteria for intervention on the tricuspid valve have focused on the degree of tricuspid annular dilatation and the severity of tricuspid regurgitant volume. The surgical decision making regarding intervention on the tricuspid valve remains obscure and historical cohort data cannot distinguish cause from effect.

“*Even if the degree of regurgitation is determined, the clinical significance and optimal therapeutic intervention (medical management vs. surgical correction) remain difficult to determine, primarily because tricuspid regurgitation is most often secondary to, or accompanied by, another disease process. The relative contribution of the regurgitant blood flow to the clinical situation may be difficult to assess in the face of right ventricular failure or elevated pulmonary arterial pressure*.” *(**1**)*.

## Introduction

The role of surgical intervention in the pathophysiology of functional tricuspid regurgitation (TR) is obscure. While moderate to severe tricuspid regurgitation is associated with high mortality (2), indications and optimal timing of operative intervention are not well-established. Significant TR is most often secondary and related to tricuspid annular dilation and leaflet tethering in the setting of RV remodeling because of pressure or volume overload (e.g., primary pulmonary hypertension or PH secondary to left-sided heart disease). Current recommendations for surgical intervention identify populations with severe TR undergoing left-sided valve surgery or patients with tricuspid annular dilatation in the absence of pulmonary hypertension and dilated cardiomyopathy (3). Recommendations for concomitant tricuspid surgery at the time of LVAD implantation are not supported by prospective clinical trials and largely reflect surgical intuition. Consensus statements consider tricuspid intervention as “generally accepted” if not recommended (4) and suggest that TV repair be considered in “carefully selected patients” (5). However, given that significant TR in the post LVAD population is associated with increased mortality (6), it is reasonable to ask whether an objective and replicable standard for tricuspid valve intervention can be identified and made operational.

## The current supposition of TR and LVAD

Tricuspid regurgitation secondary to left sided heart failure is a consequence of RV dilatation (mid-ventricular anterolateral wall), caudal displacement of the anterior papillary muscle, leaflet tethering, and valvular deformation. While there is minimal annular dilatation early in the natural history of the pressure loaded right ventricle, increasing right ventricular (RV) diastolic volume worsens the coaptation defect as the tricuspid annulus dilates along the anterolateral axis. Progressive interventricular septal shift toward the left ventricle increases LV diastolic pressure with increased RV afterload and “TR begets more TR.” Chronic volume overload results in right ventricular remodeling, variously defined by the changes in ventricular geometry and compliance which describe RV dysfunction.

Despite the reduction in RV pressure overload that accompanies implantation of a left ventricular assist device, residual TR can persist. Fixed pulmonary vascular resistance, residual mitral regurgitation, and inadequate decompression of the left ventricle (pump position, pump speed, and afterload) can all contribute to right sided atrioventricular incompetence. Acute unloading of the dilated LV causes a leftward shit of the interventricular septum, decreasing the septal contribution to RV contraction and altering RV geometry with exacerbation of antero-septal tricuspid leaflet tethering. Early RV failure after LVAD is defined by an inability to separate from cardiopulmonary bypass (e.g., inadequate LVAD filling requiring right ventricular assist device) and is likely a distinct physiology from the progressive RV failure seen in postoperative LVAD patients. Tricuspid regurgitation is common to the distorted geometry of both acute and chronic RV failure after LVAD implantation.

## What if we do nothing? The natural history of TR after LVAD

Nakanishi et al. (7) examined the prevalence and prognostic significance of residual TR in patients with more than 1 year of LVAD support. Significant residual TR—defined as a regurgitant jet > 20% of the right atrial area—was observed in ~25% of patients. While residual TR was significantly associated with mortality, there was no significant survival difference in patients with and without preoperative TR. Right ventricular fractional area change (RVFAC) and tethering distance (e.g., the distance from atrial surface of the tricuspid leaflet to the tricuspid annular plane) were improved only in patients without residual TR. Preoperative TV annular diameter, but not TV tethering, was significantly associated with residual TR. Interestingly, TV annulus diameter increased in all patients after 1 year of LVAD support, from 41.7 to 44 mm (*p* = 0.033) among patients with residual TR and from 38.7 to 41.1 mm (*p* = 0.017) among patients without residual TR. Most importantly, multivariate logistic regression identified residual MR as the most significant predictor of residual TR (OR 4.5).

In an analysis of the EUROMACS database, Veen et al. (8) observed an immediate decrease in significant TR to non-significant TR in two-thirds of patients after isolated LVAD implantation. The odds of moderate to severe TR after an LVAD decreased even further over time, becoming comparable after ~1.4 years in patients with preoperative moderate to severe TR vs. patients with none to mild TR pre-LVAD. There were also notable differences in disease etiology: post LVAD TR decreased faster in patients with idiopathic dilated cardiomyopathy compared to other diagnoses suggesting that biological differences in ventricular biology impact the efficacy of left ventricular support. While residual TR was associated with both early and late mortality, patients with significant preoperative RV dysfunction and severe TR had post implant survival and hazard ratios comparable to those patients with significant preoperative RV dysfunction and minimal TR. In a sensitivity analysis, pre-LVAD right ventricular dysfunction was identified as the driving factor on mortality regardless of the severity of pre-LVAD TR. Sensitivity analysis is an attempt to avoid the confounding effect of tricuspid regurgitation, as TR is both a consequence of and a contributor to right ventricular dysfunction.

In a single institution study, Zadok et al. (9) found that among patients with significant TR pre-LVAD, more than half (55%) ameliorated their TR severity by 6 months. Among patients with residual TR (e.g., persistence of significant regurgitant fraction) after implantation, right ventricular stroke work index (RVSWI) was significantly lower in comparison to patients whose TR resolved (242 vs. 432). A similar relationship was demonstrated for the pulmonary artery pulsatility index (PAPI) with residual TR patients having significantly less contractile reserve. In short, patients who failed to improve their TR severity grade post-surgery demonstrated worse RV systolic function as assessed by hemodynamic parameters. Other than atrial fibrillation, there were no hemodynamic or clinical markers among the pre-LVAD patients with significant TR to predict post implant residual tricuspid regurgitant disease. Interestingly, 13% of patients without significant TR at the time of LVAD implant progressed to significant TR over the course of the study (1 year followup). Again, significant post-LVAD TR was associated with mortality.

## The evidence: Bias, confounding, and questions of study design

*Confounding* is the situation in which the epidemiologic difference in the risk of the outcome between exposed (tricuspid valve intervention) and unexposed (no tricuspid surgery) can be explained by other differences in the contrasted groups (10). The vast majority of published studies on the impact of tricuspid valve repair at the time of LVAD implantation are retrospective and observational and nearly all are historical cohort studies comparing outcomes between LVAD patients with and without tricuspid valve intervention (11–15). There is statistical *confounding by indication*. “Treatment” (e.g., tricuspid intervention) is preferentially prescribed to groups of patients based on their underlying risk profile (e.g., severity of TR or annular dilatation). Consequently, patients exposed or not exposed to intervention might not be comparable, precluding any causal inference between tricuspid valve repair and outcome. This is *selection bias*, best described as a potential fundamental difference among the patients in the treatment arm (tricuspid intervention) due to the way in which patients were allocated to the treatment group.

Far more important is the question of *misclassification bias* in the published observational studies. Significant TR can—and frequently does—resolve after isolated LVAD implantation. Tricuspid valve repair in a patient with significant preoperative TR that would have resolved after isolated left ventricular unloading is *misclassified* as the tricuspid intervention is redundant, valvular intervention did not impact TR. Differential misclassification bias skews the data toward the null hypothesis (e.g., tricuspid intervention has no impact on the primary outcome), making historical cohort studies an unlikely source of information for surgical decision making.

Another confounder is the relationship between tricuspid regurgitation and RV failure. Significant tricuspid regurgitation is well-tolerated in LVAD patients without RV dysfunction (16) and RV dysfunction is found among patients with and without significant TR. While TR is treatment variable under study, RV dysfunction is the clinical variable associated with outcome.

Propensity scoring, wherein the likelihood of being exposed to the intervention (e.g., tricuspid valve surgery) is used to match patients can account for confounding. Veen et al. (17) in an examination of the EUROMACS registry used retrospective propensity scoring to compare nearly 500 patients who underwent LVAD implantation with or without tricuspid valve surgery. While hospital deaths, days on inotropic support, use of temporary RVAD support, and cumulative incidence of right heart failure were comparable in both groups, patients with tricuspid surgery had significantly longer stays in the ICU (*P* = 0.026). Despite significantly less moderate to severe TR immediately after surgery in the tricuspid intervention group, differences in the probability of TR disappeared during the follow up period suggesting that concomitant TV surgery is not associated with improved clinical outcome.

To avoid the confounding relationship between TR and ventricular function, the TVVAD trial (NCT03775759) stratified patients by pre-operative right ventricular dysfunction (none/mild vs. moderate vs. severe) at the time of randomization. Sixty patients with moderate or severe TR on pre-operative echocardiography were randomized to either LVAD implantation alone (no TVR, *n* = 28) or LVAD implantation with concomitant tricuspid valve surgery (TVR, *n* = 32). At 6 months there was no difference in the incidence of moderate or severe right heart failure (46% in the LVAD only group and 44% in the group with LVAD and concomitant tricuspid intervention).

Despite the clinical value of observational cohort studies, they provide the weakest epidemiologic evidence for causation and efficacy of intervention, as the risk of uncontrolled bias and confounding are potentially lethal flaws. Greenwood's (18) adage that he should like to shame surgeons out of “the comic opera performances which they suppose are statistics of operations” may be hyperbole, but the criticism is valid. The ecological fallacy has merit in surgical epidemiology and one cannot infer the properties of an individual from the average response of the group (19). Even if the appropriate level of aggregated data were identified, surgeon specific differences significantly impact the validity of retrospective observational studies (20) and it is unlikely that historical cohort data could inform patient—specific surgical decision making.

## Is it the tricuspid valve… are we measuring the wrong thing?

The goal of valve surgery is the preservation of ventricular function and intervention on the tricuspid valve is premised upon the impact a reduction in TR will have on progressive RV dysfunction and subsequent RV failure. But, in the absence of structural valve disease, is it reasonable to expect intervention on the tricuspid annulus to impact ventricular biology? Does unrepaired TR drive ventricular remodeling and subsequent RV dysfunction? Is functional TR a consequence of RV failure, a mechanism of RV failure, or both? The short answer is we do not know.

The role of tricuspid annular dilatation, tricuspid regurgitation, and RV dysfunction is problematic for surgeons. Annular pathology seems such a correctable target for surgical intervention, particularly given the association between tricuspid annular diameter (>40 mm) and late right heart failure (21). However, recent studies of patients undergoing guideline—directed repair of functional TR (annular diameter > 40 mm independent of TR severity) at the time of mitral surgery demonstrate no differences in survival and the incidence of “late TR” is low in patients with unrepaired mild TR (22). While Gammie et al. (23) recently demonstrated a lower incidence of progression to TR in patients who underwent tricuspid annuloplasty at the time of mitral valve repair, preliminary data do not address the role of recurrent mitral regurgitation on the subsequent evolution of tricuspid insufficiency. Importantly, tricuspid annular dilatation was not a predictor of progressive TR in the absence of baseline regurgitation suggesting that annular dilatation alone is not a viable criterion for surgical decision-making. In the absence of tricuspid repair, moderate to severe TR after MVR did not predict clinical outcomes or performance standards at 2 years.

There are strong theoretical arguments for the surgical correction of TR, but the physiological studies upon which the intervention is premised also demonstrate the over-riding importance of preload and afterload in determining RV stroke volume and ventricular performance. Nearly one fourth of our patients have moderate to severe MR after isolated cfLVAD and this persistent RV afterload is associated with an increased incidence of right heart failure (RAP > 14 mmHg, cardiac index <2.2 L/min/m^2^, and need for inotropic support at 6 months), higher mean pulmonary artery pressures, and elevated pulmonary capillary wedge pressure (24). There were no differences in LVAD parameters between the MR severity groups and significant residual MR did not predict functional TR after isolated LVAD despite the MR severity dependent association with progressive RV dysfunction. Similar findings have been reported by the Michigan group (25) where postoperative cfLVAD MR severity independently correlated with the incidence of RV failure. Here, however, MR severity had a positive correlation with TR severity and TV repair to improve valve competence was associated with worsened RV function.

While persistent MR after LVAD is a consistent marker of progressive RV failure (26), residual TR is not (27). In our experience the prevalence of significant residual MR after LVAD is similar between the groups with insignificant and significant TR, suggesting residual left sided failure is not the only etiology. Patients with significant residual TR after LVAD implantation frequently demonstrate decreased right ventricular stroke work index (RVSWI) and pulmonary artery pulsatility (PAPI)—both specific measures of RV function. If the rationale for tricuspid repair is the preservation of RV function, then functional metrics of RV performance should correlate with the severity of tricuspid regurgitation. While there is no clearly defined and broadly accepted definition of RV dysfunction or RV failure, we have found pulmonary artery pulsatility index (PAPi) a useful predictor of presumed intrinsic RV dysfunction (28). PAPi is the only measure of right heart physiology that is known to correlate with RV specific myocyte dysfunction as measured by calcium sensitivity and contractile reserve (29). A lower pulmonary artery pulsatility score was associated with more severe TR in a *post-hoc* analysis of the ESCAPE trial and PAPi—but not RAP:PCWP ratio or RVSWI—was a significant predictor of mortality by multivariable Cox regression analysis (30). Pulmonary artery pulsatility index (PAPi < 1.8) is associated with various measures of right heart failure after LVAD implantation (31) and pre implantation PAPi score is a predictor of subsequent RVF after LVAD (32). Even in patients without pulmonary hypertension, significant TR is associated with lower PAPi scores (right ventricular dysfunction) and worse survival (33). PAPi scores might provide a more consistent marker for RV reverse remodeling and allow clinical trial design that is focused on the mechanisms that result from surgical intervention (annuloplasty) rather than the degree of improvement in clinical outcome.

## What we think we know

Residual or recurrent TR after LVAD implantation—particularly that associated with progressive RV dysfunction—is a poor prognosticator and a consistent marker of patient mortality (6). Numerous studies suggest that concomitant TV intervention is not associated with freedom from RV dysfunction and there is no consensus on the indication for TV intervention at the time of LVAD implant (annular dilatation of >40 mm or severity of regurgitation). The significant pre-operative TR common to end stage heart failure improves (and frequently resolves) in the majority of patients after LVAD implantation independent of intervention on the tricuspid valve (7–9). Intervention on the tricuspid valve at the time of LVAD has never demonstrated a survival advantage and concomitant TV procedures are associated with increased morbidity and mortality in a stratified analysis of the INTERMACS database (12). While concomitant TV surgery has been demonstrated to improve LVAD filling and hemodynamics (15), tricuspid annuloplasty does not impact the incidence or progression of late RV failure (27). Concomitant tricuspid surgery has a significant fail rate (14) and a small but persistent subset of patients (10–15%) without pre-operative TR develop TR over time (8).

Atrial fibrillation associated TR is a distinct group of LVAD patients in which concomitant tricuspid valve surgery may be warranted. The Michigan group has recently demonstrated that functional MR related to atrial fibrillation and characterized by a dilated left atrium had excellent survival and low recurrence after annuloplasty (34). Importantly, patients with “atrial MR” had preserved left ventricular end-diastolic volumes (LVEDV < 5 cm). Answer et al. (35) have argued for including atrial fibrillation in the surgical decision making on tricuspid procedures during LVAD implantation.

## Unanswered questions: Is there a rationale for concomitant tricuspid repair?

The role of surgery in patients with a dilated annulus and minimal TR remain controversial, as does the role for intervention in patients with significant TR and preserved annular dimensions. Annuloplasty of the dilated annulus with severe TR may reduce the physiologic impact of RV dysfunction but demonstrates no consistent relationship to a documented reversal of right ventricular remodeling that is thought to impact long-term survival. At present, we cannot identify an LVAD patient “at risk” for severe post-implant TR and there is no reason to believe that “prophylactic” reduction annuloplasty might impact the incidence of progressive disease. (e.g., downsizing mitral annuloplasty does reduce left ventricular end diastolic volume and improve LV ejection fraction but demonstrates no improvement in survival when compared to optimized medical therapy) (36).

Given the high incidence of recurrent TR after annuloplasty, is repair the wrong approach? Would valve replacement alter the mechanics of RV dysfunction and subsequent RV failure? AICD leads and biventricular pacing wires are nearly ubiquitous in the end stage heart population and “pinning” of tricuspid leaflets by trans-annular EP device leads is a common observation (25% in our patient population). Annuloplasty is unlikely to significantly impact the tethered leaflet. Is reduction annuloplasty with a flexible band or remodeling annuloplasty with a rigid ring relevant to the conversation regarding concomitant surgery and TV repair? Does annuloplasty ring size impact durability and ventricular pathophysiology, or does a “one size 28 mm reduction annuloplasty fit all”? Given the importance of RV geometry and the impact of pump speed on septal and posterior leaflet displacement, is preservation of the pericardium and passive ventricular constraint more important than preservation of annular dimensions? Many of us embrace the reduction in RV failure seen with the thoracotomy approach as more than case selection bias (37). Are the known gender differences in the incidence of TR significant to surgical decision making (38)? Most importantly, does intervention on the tricuspid valve impact RV function and contribute to reverse remodeling?

## Any conclusions?

Surgeons looking to the aggregate data of historical population studies for surgical decision making will be frustrated by differences in study design, variable definitions and descriptions of RV dysfunction, and most significantly by the remarkable complexity of right ventricular failure. Despite enormous amounts of data, there is little information, and even less knowledge as to the “correct” surgical decision. What is clear is that no “once size fits all” approach to TR at the time of LVAD implantation will be effective therapy for all patients. While there may be patients who would benefit from TV procedures at the time of LVAD implant, defining a population cohort for whom evidence based data can recommend intervention seems unlikely given the dynamic complexity of functional TR. It is more likely that biomarker and functional imaging data will define a patient cohort in which TV intervention is ill advised and unlikely to contribute to reverse remodeling. As noted by McGee (39), effective heart failure surgery is being able to discriminate the patients that will improve from those that will not benefit or be potentially harmed from the surgical procedure. Perhaps the question of concomitant surgery is itself superfluous. Transcatheter approaches to the tricuspid valve are rapidly evolving and it is likely that percutaneous intervention prior to or after LVAD implantation will allow more nuanced and temporally appropriate patient specific therapies (40). In the interim, we are left with imaging, statistical inference, and the too often disregarded judgement that comes with clinical experience.

## Author contributions

The author confirms being the sole contributor of this work and has approved it for publication.

## Conflict of interest

The author declares that the research was conducted in the absence of any commercial or financial relationships that could be construed as a potential conflict of interest.

## Publisher's note

All claims expressed in this article are solely those of the authors and do not necessarily represent those of their affiliated organizations, or those of the publisher, the editors and the reviewers. Any product that may be evaluated in this article, or claim that may be made by its manufacturer, is not guaranteed or endorsed by the publisher.
